# Assessment of clustering techniques to support the analyses of soybean seed vigor

**DOI:** 10.1371/journal.pone.0285566

**Published:** 2023-08-25

**Authors:** Eduardo R. de Oliveira, Pedro H. Bugatti, Priscila T. M. Saito

**Affiliations:** 1 Department of Computing, Federal University of Technology - Parana, Cornelio Procopio, PR, Brazil; 2 Department of Computing, Federal University of Sao Carlos, Sao Carlos, SP, Brazil; National University of Science and Technology, PAKISTAN

## Abstract

Soy is the main product of Brazilian agriculture and the fourth most cultivated bean globally. Since soy cultivation tends to increase and due to this large market, the guarantee of product quality is an indispensable factor for enterprises to stay competitive. Industries perform vigor tests to acquire information and evaluate the quality of soy planting. The tetrazolium test, for example, provides information about moisture damage, bedbugs, or mechanical damage. However, the verification of the damage reason and its severity are done by an analyst, one by one. Since this is massive and exhausting work, it is susceptible to mistakes. Proposals involving different supervised learning approaches, including active learning strategies, have already been used, and have brought significant results. Therefore, this paper analyzes the performance of non-supervised techniques for classifying soybeans. An extensive experimental evaluation was performed, considering (9) different clustering algorithms (partitional, hierarchical, and density-based) applied to 5 image datasets of soybean seeds submitted to the tetrazolium test, including different damages and/or their levels. To describe those images, we considered 18 extractors of traditional features. We also considered four metrics (accuracy, FOWLKES, DAVIES, and CALINSKI) and two-dimensionality reduction techniques (principal component analysis and t-distributed stochastic neighbor embedding) for validation. Results show that this paper presents essential contributions since it makes it possible to identify descriptors and clustering algorithms that shall be used as preprocessing in other learning processes, accelerating and improving the classification process of key agricultural problems.

## 1 Introduction

Soy is the fourth most cultivated bean globally and the main product in Brazilian agriculture. In 2021/22, Brazil estimates a production record of 142,009 million tons of soybeans. This value was obtained from the increase of 3.4% of the cultivated area compared to 2020/21, and 4.3% of increased productivity per hectare [1].

Those numbers would only be achieved with scientific advances and the availability of new technologies in the productive sector [2]. Among advances, mechanization, soil management, and prevention solutions for pests and diseases stand out.

Vigor tests are a way to obtain good information for consequent scientific advances. They are commonly used to find quality differences among bean lots during storage or after sowing, presenting the best and highlighting their planting conditions [3]. One of these tests is the tetrazolium test. It determines the vigor of soybean lots and offers information on the causes of quality reduction, identifying mechanical damages, moisture, and bug deterioration [4].

However, the analysis for damage classification is done visually by a specialist. This task may be unavailable considering the number of samples in a lot. The damage analysis of soybeans submitted to the tetrazolium test is a slow and tiresome method when performed manually since it is a visual task and requires hours of work [4].

Efforts in the literature [3, 5–8] show that supervised learning techniques for damage classification after the tetrazolium test could present good results. Therefore, to increase productivity, papers such as [5–8] propose active learning techniques (supervised classification) for this type of analysis. Despite having significant results, these papers focus on supervised learning techniques.

Non-supervised learning techniques are rarely explored in this context. Some active learning strategies that use grouping techniques as pre-processing consider, for example, the k-means algorithm. However, an extensive experimental evaluation is not done, comparing the performances of different clustering algorithms. Therefore, this paper investigates different non-supervised techniques to contribute to the research of unsupervised learning and active learning strategies applied to the classification of soybean damages.

In summary, this paper aims to perform an extensive experimental evaluation, considering different unsupervised techniques to improve the classification process of soybeans subjected to the tetrazolium test. Our **contributions** are fourfold: i) organization of soybean image groups; ii) extraction and selection of well-suited features that describe the images’ clustering; iii) extensive analyses and performance evaluation of different clustering techniques; iv) novel comparative analyses and validation of the obtained results considering different image datasets, clustering, descriptors techniques and different metrics to a vital agricultural problem.

## 2 Materials and methods

This section presents image description techniques and unsupervised learning methods used in this paper. We also present different methods for clustering evaluation and graphical/visualization results.

### 2.1 Descriptor learning

It is necessary to capture the different damage patterns that separate into specific classes to classify a set of soybean images. These descriptors consider different visual properties based on color, shape, and texture. Therefore, each descriptor extracts and generates a feature vector (numeric values), describing the images.

#### 2.1.1 Feature extraction

There are several feature extractors in the literature. Color-based extractors are widely used, especially for natural image classification. Some extractors consider the color histogram [9], which describes the global image content according to the pixel percentage of each color.

Other examples of color-based extractors are: Auto Color Correlogram (ACC) [10], Border/Interior Pixel Classification (BIC) [11], Color and Edge Directivity Descriptor (CEED) [12], Global Color Histogram (GCH) [13], Local Color Histogram (LHC) [14], Reference Color Similarity (RCS) [15], among others.

Texture properties can also represent images since they have information about luminosity, spatial distribution, and structural arrangement of the surface relating to the neighboring region. Gabor filters [16], Haralick descriptors [17], Local Binary Pattern (LBP) [18], Moments [19], First Order Statistics (MPO), First Order Statistics w/ color (MPOC), Pyramid Histogram of Oriented Gradients (PHOG) [20], Tamura [21] are examples of texture-based extractors.

Some extractors combine different types of features, like the Fuzzy Color and Texture Histogram (FCTH) [22] and the Join Composite Descriptor (JCD) (FCTH + CEDD) extractors that combine characteristics based on color and texture to describe images.

### 2.2 Machine learning

Machine learning is an area of artificial intelligence that proposes the development of systems capable of learning a specific pattern or behavior automatically using examples or experience. To do so, different supervised and unsupervised learning approaches can be considered.

#### 2.2.1 Supervised classification

In supervised classification, the learning process is performed through a set of (training) previously labeled data [23]. An oracle or specialist needs to annotate samples that best represent a class in this process. Then, the algorithm, after training, can present a better result [24]. Examples of supervised learning methods from the literature are: Random Forest (RF) [25], Support Vector Machines (SVM) [26], and Optimum-Path Forest (OPF) [27].

However, the supervised learning technique presents challenges: sample unbalancing in dataset classes, noises related to imperfect data or outliers, overfitting, underfitting, and missing values for some features. These challenges directly depend on the data used in learning training.

#### 2.2.2 Unsupervised classification

The unsupervised classification aims to find data clusters in multidimensional feature space according to specific similarity criteria. Using similarity relations, similar samples are grouped in the same cluster. Therefore, it is possible to describe the inherent characteristics of each cluster made from the grouping process, enabling a better understanding of the clustered data. Several clustering methods can be separated into categories: partitional, hierarchical, and density-based.

Partitional methods make *k* data partitions (clusters). This model uses an iterative relocation technique to improve partitioning from initial partitioning. The main method of this category is the well-known k-means. Proposed in [28], it is one of the most popular clustering algorithms. This method considers four phases: initialization, clustering, centroids’ movement, and optimization. In the initialization, random samples are defined as centroids (i.e., central cluster points). The clustering phase calculates the distance (e.g., Euclidean) between all samples and centroids. Next, each sample is assigned to the cluster where the centroid presents the shortest distance. After the clustering, the centroids’ movement phase calculates the sample mean of each cluster. Finally, samples closer to the mean are assigned as new centroids. Therefore, the optimization phase performs the clustering phases and centroids’ movement repeatedly until the central values of the clusters stabilize, reaching the final clusters.

Like k-means, the k-medoids algorithm [29] has the same phases. However, in the centroids’ movement phase, it is not the nearest sample that is defined as the centroid (i.e., ghost sample), but one of the samples (called medoid) of the clustering that minimizes the distance for all samples. Compared to k-means, this method is less susceptible to noise, since the outliers have little effect on the medoid choice. CLARANS [30] and FCM [31] are other examples of partitional algorithms.

Hierarchical methods are divided into agglomerative and divisive. These methods build (agglomerative) or split (divisive) a binary tree, in which nodes are samples of the data clustering, and connections are made based on data distance (dissimilarity). AGNES [32], CURE [33], and ROCK [34] are examples of hierarchical algorithms.

Density-based methods perform the clustering according to density established through input parameters. These parameters dictate the minimum density necessary to form a cluster. The main technique of this category is the Density-Based Spatial Clustering of Application with Noise (DBSCAN) [35]. This algorithm defines that a cluster has central points and border points. Central points are those that, given a radius *r*, have a defined (MinPts) minimum neighboring points (samples). Border points do not have minimum neighboring points, but one of the neighbors must be a central point. This technique finishes when no new points can be assigned to a cluster. This paper also considers the density-based algorithm named OPTICS [36].

#### 2.2.3 Clustering evaluation

Two metrics are considered to analyze clustering: those based on the true label of samples; and those not using this information. Although our datasets present the samples’ true label, we also considered metrics that do not consider such information to reach a broader and better analysis. These metrics are Fowlkes-Mallows (FOWLKES) [37], Davies-Bouldin (DAVIES) [38] and Calinski-Harabasz (CALINSKI) [39].

**Fig 1:**
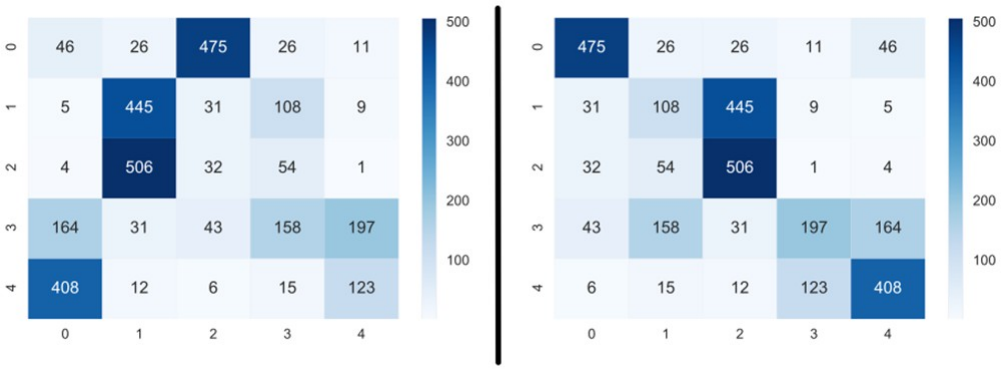
Matrix reorganization to reach the best accuracy from a given cluster: (a) before and (b) after reorganization.

Another well-known metric is accuracy, and it gives the correctness samples percentage. It is calculated by the number of correctly clustered samples divided by the total number of samples. Clustering techniques are not committed to correctly defining labels, only to cluster samples. As a result, clusters may or may not adequately represent different classes. Therefore, accuracy calculation is based on a method that organizes the assigned labels and finds the highest accuracy possible [40]. To do so, it is used data from the confusion matrix, in which lines comprises true labels and columns are clusters. The goal is to find the column reorganization that generates the best accuracy result (i.e., an organization where the biggest values of each line are on the main diagonal of the matrix). Fig 1(a) and 1(b) present (pre and post) matrix reorganization examples, respectively.

Another metric that uses true label knowledge is FOWLKES [37], which is defined as the geometric mean of precision and recall. Eq 1 presents the metric formula, where TP corresponds to the number of true positives (samples correctly grouped with label *C*, that are part of *C*), and FN represents the number of false negatives (samples that should be grouped with *C* label, but were not). The range of the Fowlkes metric is between 0 and 1. The higher the value, the greater the similarity between the found clusters and the ground-truth classification.

$$\begin{array}{c} FOWLKES=\frac{VP}{\sqrt{\left(TP+FP)+(TP+FN\right)}} \end{array}$$ (1)

When the ground-truth labels of the samples are unknown, metrics are employed to evaluate the quality of obtained clustered. These metrics are generally based on cohesion and separation measures. Cohesion refers to the distance between samples of the same cluster, and separation indicates the distance between clusters [41].

The DAVIES metric [38] compares the distance between clusters with their sizes to indicate how good the separation between them is. Eq 2 formally defines the DAVIES metric.

$$\begin{array}{c} DAVIES=\frac{1}{k}\sum_{i=1}^{k}\underset{i\neq j}{max}R_{ij} \end{array}$$ (2)

where *k* represents the total number of clusters and *R*_*ij*_ comprises the comparison between two clusters. *R*_*ij*_ calculation is presented by Eq 3.

$$\begin{array}{c} R_{ij}=\frac{s_{i}+s_{j}}{d_{ij}} \end{array}$$ (3)

where *s*_*i*_ represents the mean distance of each sample of the group *i* to its centroid, and *d*_*ij*_ is the distance between *i* and *j* clusters’ centroids. Results range from 0 to 1, and lower values indicate better groupings.

CALINSKI is another metric that does not use true label knowledge. In this case, high values indicate better clustering definitions (i.e., dense and well-separated clusters). Its value is generated by the dispersion sum ratio between clusters and the internal dispersion of each cluster [39].

To a dataset *E* with size *n*_*E*_ and *k* clusters, the CALINSKI definition is presented by Eq 4, where *tr*(*B*_*k*_) represents the dispersion matrix trace between clusters, and *tr*(*W*_*k*_) is the cluster internal dispersion matrix trace (defined by Eqs 5 and 6). In this case, *C*_*i*_ represents the *i* cluster’s samples set, *c*_*i*_ is the *i*-th cluster center, *c*_*e*_ is the *e* center, and *n*_*i*_ is the number of samples from the *i*-th cluster.

$$\begin{array}{c} CALINSKI=\frac{tr(B_{k})}{tr(W_{k})}*\frac{n_{E}-k}{k-1} \end{array}$$ (4)

$$\begin{array}{c} W_{k}=\sum_{i=1}^{k}\sum_{x\in C_{i}}\left(x-c_{i}\right){\left(x-c_{i}\right)}^{T} \end{array}$$ (5)

$$\begin{array}{c} B_{k}=\sum_{i=1}^{k}n_{i}\left(c_{i}-c_{e}\right){\left(c_{i}-c_{e}\right)}^{T} \end{array}$$ (6)

#### 2.2.4 Dimensionality reduction

Descriptors presented in Section 2.1.1 describe images in multiple-dimension vectors. Therefore, the graphic visualization of samples’ real values is impossible. In order to solve this problem, dimensionality reduction methods are applied to reduce samples in two or three dimensions.

Dimensionality reduction techniques (or feature reduction) are separated into feature selection methods and feature transformation methods [42]. Selection methods are based on evaluating which sample features are the most important for a better representation. Transformation methods use all sample features to calculate a new representation and are more suitable for graphic visualization.

Some feature reduction techniques based on transformation use linear and nonlinear functions. Principal Component Analysis (PCA) [43] is a technique that performs linear data mapping to a reduced dimension. Therefore, in the reduced dimension, data variation is maximized. Another one is the t-distributed Stochastic Neighbor Embedding (t-SNE) [44], a nonlinear technique that calculates the probability of features’ similarity and minimizes the divergence between the reduced and the original sample probability.

## 3 Proposed methodology

**Fig 2:**
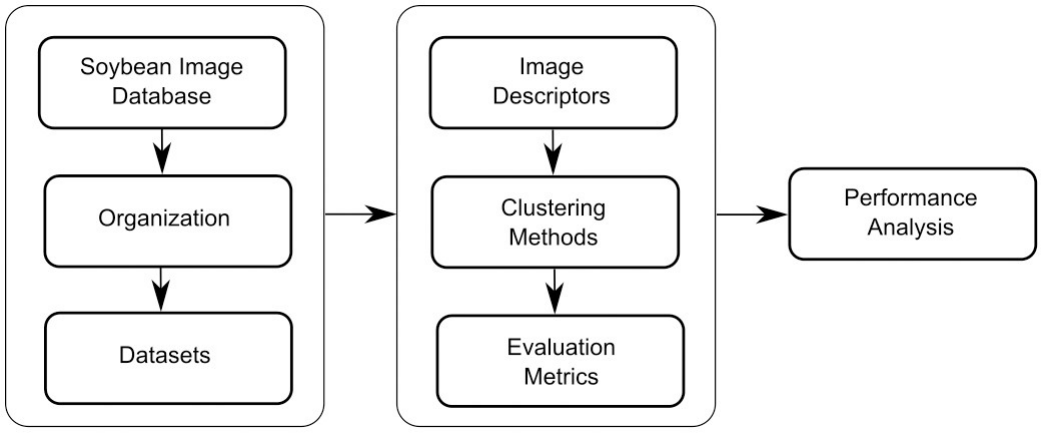
Pipeline of our proposed methodology.

This section presents the proposed methodology for soybean image classification, describing the steps of each process. We present the description of the soybean image datasets (Section 3.1), experimental scenarios (Section 3.2), image descriptors, and algorithm configuration. Fig 2 illustrates the pipeline of our proposed methodology.

Soybean images can be organized in many ways, resulting in different datasets for experimental evaluation. We removed the images with stain and clipping problems in this stage. Section 3.1 presents more details about the organized datasets.

We extracted features for each organized dataset. In this stage, each soybean image is represented by a feature vector, considering descriptors presented in Section 2.1.

From the feature extraction process, clustering methods (presented in Section 2.2.2) are evaluated through different metrics (mentioned in Section 2.2.3). Therefore, it is possible to analyze the performance of each algorithm and its configurations in soybean classification.

### 3.1 Description of the datasets

After the tetrazolium test, analysts perform a visual process to identify and label each soybean portion (containing two internal parts and two external parts) into classes. The class is defined by the damage type and its severity level.

In [8], two image acquisitions were performed in a real soybean seed enterprise, where 1400 images were obtained from the first one and 1358 from the second one. Then, we generated different subsets from both image acquisitions in our work.

To do so, we consider each soybean portion labeled separately, given that we observed better performance from this scenario in [8]. This paper identifies the datasets as *D*_1_-*D*_5_. *D*_4_ and *D*_5_ ones are reorganizations of the *D*_3_ dataset. Tables 1 to 5, respectively, present a description and quantity of samples in each dataset class.

**Table 1:** Classes’ descriptions, samples’ distribution for each class of dataset *D*_1_, and their respective severity levels.

Classes	Description	Samples
OXE	Perfect External Portion	483
OXI	Perfect Internal Portions	499
2UE	Humidity Damage External Portion—Level 2	23
2UI	Humidity Damage Internal Portion—Level 2	7
3ME	Mechanic Damage External Portion—Level 3	32
3MI	Mechanic Damage Internal Portion—Level 3	28
3PE	Stinkbug Damage External Portion—Level 3	78
3PI	Stinkbug Damage Internal Portion—Level 3	36
3UE	Humidity Damage External Portion—Level 3	36
3UI	Humidity Damage Internal Portion—Level 3	47

**Table 2:** Classes’ descriptions, samples’ distribution for each class of dataset *D*_2_, and their respective severity levels.

Classes	Description	Samples
OXE	Perfect External Portion	306
OXI	Perfect Internal Portion	374
3ME	Mechanic Damage External Portion—Level 3	4
3MI	Mechanic Damage Internal Portion—Level 3	5
3PE	Stinkbug Damage External Portion—Level 3	18
3PI	Stinkbug Damage Internal Portion—Level 3	17
3UI	Humidity Damage Internal Portion—Level 3	9

**Table 3:** Classes’ descriptions, samples’ distribution for each class of dataset *D*_3_, and their respective severity levels.

Classes	Description	Samples
OXE	Perfect External Portion	789
OXI	Perfect Internal Portion	873
2UE	Humidity Damage External Portion—Level 2	23
2UI	Humidity Damage Internal Portion—Level 2	7
3ME	Mechanic Damage External Portion—Level 3	36
3MI	Mechanic Damage Internal Portion—Level 3	33
3PE	Stinkbug Damage External Portion—Level 3	96
3PI	Stinkbug Damage Internal Portion—Level 3	53
3UE	Humidity Damage External Portion—Level 3	36
3UI	Humidity Damage Internal Portion—Level 3	56

**Table 4:** Classes’ descriptions and samples’ distribution for each class of dataset *D*_4_.

Classes	Description	Samples
XE	Perfect External Portion	789
XI	Perfect Internal Portion	873
UE	Humidity Damage External Portion	59
UI	Humidity Damage Internal Portion	63
ME	Mechanic Damage External Portion	36
MI	Mechanic Damage Internal Portion	33
PE	Stinkbug Damage External Portion	96
PI	Stinkbug Damage Internal Portion	53

**Table 5:** Classes’ descriptions and samples’ distribution for each class of dataset *D*_5_.

Classes	Description	Samples
X	Perfect Portion	1662
U	Humidity Damage Portion	122
M	Mechanic Damage Portion	69
P	Stinkbug Damage Portion	149

*D*_1_ and *D*_2_ are, respectively, the first and second acquisitions, in which we organized samples with different damages and their severity level between 0 and 3. However, considering the *D*_1_ dataset, images that presented stains and cutting problems were removed in the organization stage of the datasets since they can impact algorithms’ learning process and performance.

*D*_3_ dataset is the aggregation between the *D*_1_ and the *D*_2_ ones. Since *D*_3_ presents all possible samples, the *D*_4_ and the *D*_5_ datasets were created from it. *D*_4_ is a reorganization of the *D*_3_ dataset where we removed the severity levels (i.e., only damage types are considered). In the *D*_5_ dataset, both severity levels and the differentiation of internal and external portions were removed. These new datasets were created to measure how the change in the samples’ number and classes impact experiments. Table 6 summarizes each dataset regarding their number of classes and samples.

**Table 6:** Number of classes and samples for each dataset.

Image Datasets	Classes	Samples
*D*_1_	10	1269
*D*_2_	7	733
*D*_3_	10	2002
*D*_4_	8	2002
*D*_5_	4	2002

### 3.2 Description of the scenarios

In order to perform experiments, we defined different scenarios. First, we extract features from the datasets based on the image descriptors shown in Table 7 and described in Section 3.1.

**Table 7:** Image descriptors used to extract features from each soybean seed image, and their respective feature vector dimensionality.

Descriptor	Description	Category	#features
ACC	Auto Color Correlogram	Color	768
BIC	Border/Interior Classification	Color	128
CEDD	Color and Edge Directivity Descriptor	Color	144
FCTH	Fuzzy Color and Texture Histogram	Color-Texture	192
GABOR	Gabor Texture Features	Texture	60
GCH	Global Color Histogram	Color	255
HARACOLOR	Haralick Color	Color	14
HARAFULL	Haralick Full	Color-Texture	14
HARALICK	Haralick	Texture	14
JCD	Join Composite Descriptor	Color-Texture	336
LBP	Local Binary Patterns	Texture	256
LCH	Local Color Histogram	Color	135
MOMENTS	Moments	Texture	4
MPO	First Order Measures	Texture	6
MPOC	Firs Order Measures Color	Color-Texture	18
PHOG	Pyramid Histogram of Oriented Gradients	Texture	40
RCS	Reference Color Similarity	Color	77
TAMURA	Tamura Descriptor	Texture	18

After the description process, different clustering algorithms were applied and analyzed through different metrics (see Sections 2.2.3 and 2.2.4). Table 8 shows the clustering methods considered in our work.

**Table 8:** Clustering methods used in our experiments.

Method	Description
AGNES	Agglomerative Nesting
CLARANS	Clustering Large Applications based on Randomized Search
CURE	Clustering using Representatives
DBSCAN	Density Based Spatial Clustering of Application with Noise
FCM	Fuzzy-c-Means
K-Means	K-Means
K-Medoid	K-Medoid
OPTICS	Ordering Points to Identify the Clustering Structure
ROCK	Robust Clustering Using Links

According to the literature standard, default values were used as input parameters for clustering methods. In these cases, the number of classes was used.

As presented in Section 2.2.2, density-based algorithms need the radius and the minimum of neighbor’s points as a parameter. We used the technique presented in [35] to set the radius value. We performed a parametric analysis to define the minimum of the neighbor’s points. To do so, multiple experiments were performed, changing the parameter value (i.e., grid-search). Then, the value was chosen in which the resulting clustering number was the closest to the total of classes.

To define the accuracy, we performed a clustering label organization method [40], as presented in Section 2.2.3.

## 4 Results and discussion

The accuracy results make it possible to perform the first performance analysis. Tables 9 to 13 present accuracy results obtained by each descriptor and clustering method from each dataset (*D*_1_-*D*_5_, respectively).

**Table 9:** Accuracy results obtained by each descriptor and clustering method from the dataset *D*_1_. The bold values correspond to the best clustering methods for each descriptor. The underlined values highlight the descriptor that reached the best performance for each clustering algorithm. The asterisk value represents the best combination (descriptor and clustering algorithm). It is also presented the mean accuracies considering all descriptors and clustering methods.

–	AGNES	CLARANS	CURE	DBSCAN	FCM	KMEANS	KMEDOIDS	OPTICS	ROCK	Mean Acc.	Std
ACC	0,578	0,286	0,711	0,704	0,309	0,336	0,491	0,704	**0,745**	0,540	0,190
BIC	0,704	0,370	**0,744**	0,716	0,271	0,318	0,502	0,716	0,388	0,525	0,195
CEDD	0,630	0,337	**0,737**	0,390	0,423	0,331	0,664	0,390	0,394	0,447	0,155
FCTH	0,723	0,422	**0,733**	0,537	0,350	0,451	0,644	0,537	0,723	0,569	0,144
GABOR	0,404	0,283	0,473	0,394	0,260	0,281	0,289	0,394	**0,552**	0,370	0,100
GCH	0,697	0,405	*0,768	0,714	0,323	0,316	0,537	0,714	0,386	0,540	0,186
HARACOLOR	0,582	0,377	0,652	0,703	0,322	0,360	0,383	0,703	**0,722**	0,534	0,140
HARAFULL	**0,544**	0,258	0,534	0,391	0,297	0,319	0,298	0,398	0,390	0,381	0,170
HARALICK	0,471	0,293	0,589	0,385	0,350	0,366	0,355	0,393	**0,733**	0,437	0,102
JCD	0,721	0,316	**0,733**	0,392	0,395	0,389	0,650	0,392	0,395	0,487	0,164
LBP	0,277	0,195	0,341	0,391	0,169	0,191	0,198	0,391	**0,397**	0,283	0,098
LCH	0,567	0,366	0,392	0,583	0,407	0,341	**0,603**	0,583	0,394	0,471	0,109
MOMENTS	0,630	0,429	**0,724**	0,723	0,267	0,319	0,362	0,723	**0,724**	0,545	0,197
MPO	0,608	0,362	0,600	0,553	0,316	0,330	0,326	0,553	**0,685**	0,482	0,146
MPOC	0,597	0,287	**0,717**	0,389	0,261	0,300	0,301	0,389	0,407	0,405	0,155
PHOG	0,437	0,243	0,396	0,394	0,363	0,278	**0,528**	0,394	0,396	0,381	0,083
RCS	0,593	0,371	0,723	0,701	0,268	0,354	0,477	0,701	**0,746**	0,548	0,184
TAMURA	**0,536**	0,275	0,392	0,394	0,285	0,284	0,496	0,394	0,390	0,383	0,092
Mean	0,572	0,326	0,609	0,525	0,313	0,326	0,450	0,526	0,531	-	

**Table 10:** Accuracy results obtained by each descriptor and clustering method from the dataset *D*_2_. The bold values correspond to the best clustering methods for each descriptor. The underlined values highlight the descriptor that reached the best performance for each clustering algorithm. The asterisk value represents the best combination (descriptor and clustering algorithm). It is also presented the mean accuracies considering all descriptors and clustering methods.

–	AGNES	CLARANS	CURE	DBSCAN	FCM	KMEANS	KMEDOIDS	OPTICS	ROCK	Mean Acc.	Std
ACC	0,776	0,523	**0,853**	0,842	0,398	0,517	0,437	0,840	0,502	0,632	0,191
BIC	0,681	0,467	**0,920**	0,881	0,409	0,458	0,608	0,880	0,509	0,646	0,203
CEDD	0,753	0,516	**0,920**	0,790	0,444	0,466	0,783	0,854	0,512	0,671	0,184
FCTH	0,653	0,592	*0,926	0,508	0,541	0,571	0,606	0,508	0,502	0,601	0,133
GABOR	0,658	0,400	0,696	**0,853**	0,416	0,430	0,477	0,853	0,759	0,616	0,188
GCH	0,778	0,468	**0,920**	0,873	0,395	0,386	0,745	0,873	0,505	0,660	0,220
HARACOLOR	0,588	0,367	0,675	0,844	0,373	0,405	0,412	0,859	**0,888**	0,601	0,222
HARAFULL	0,630	0,456	0,628	**0,636**	0,392	0,389	0,357	0,636	0,613	0,526	0,124
HARALICK	0,573	0,427	0,630	0,465	0,420	0,435	0,426	0,521	**0,905**	0,533	0,158
JCD	0,750	0,409	**0,917**	0,866	0,405	0,515	0,626	0,859	0,506	0,651	0,203
LBP	0,416	0,237	0,411	0,505	0,239	0,257	0,286	0,492	**0,506**	0,372	0,117
LCH	0,741	0,472	0,513	0,527	0,469	0,414	**0,742**	0,580	0,012	0,497	0,215
MOMENTS	0,614	0,355	0,675	**0,844**	0,385	0,409	0,420	0,844	0,817	0,596	0,208
MPO	0,636	0,416	0,584	0,546	0,426	0,425	0,383	**0,681**	0,643	0,527	0,115
MPOC	0,540	0,411	**0,802**	0,523	0,344	0,381	0,435	0,520	0,538	0,499	0,135
PHOG	**0,602**	0,398	0,509	0,516	0,371	0,339	0,416	0,572	0,509	0,470	0,092
RCS	0,576	0,412	**0,917**	0,842	0,351	0,445	0,408	0,865	**0,917**	0,637	0,244
TAMURA	**0,559**	0,368	0,512	0,510	0,349	0,392	0,442	0,510	0,510	0,461	0,076
Mean	0,640	0,427	0,723	0,687	0,396	0,424	0,501	0,708	0,592	-	

**Table 11:** Accuracy results obtained by each descriptor and clustering method from the dataset *D*_3_. The bold values correspond to the best clustering methods for each descriptor. The underlined values highlight the descriptor that reached the best performance for each clustering algorithm. The asterisk value represents the best combination (descriptor and clustering algorithm). It is also presented the mean accuracies considering all descriptors and clustering methods.

–	AGNES	CLARANS	CURE	DBSCAN	FCM	KMEANS	KMEDOIDS	OPTICS	ROCK	Mean Acc.	Std
ACC	0,549	0,324	0,654	0,575	0,312	0,340	0,582	0,576	**0,784**	0,522	0,163
BIC	**0,504**	0,411	0,486	0,429	0,280	0,313	0,409	0,429	0,434	0,410	0,073
CEDD	**0,740**	0,315	0,436	0,433	0,364	0,326	0,476	0,433	0,437	0,440	0,125
FCTH	0,682	0,392	**0,785**	0,431	0,335	0,404	0,477	0,431	0,432	0,485	0,148
GABOR	0,325	0,297	0,322	**0,487**	0,219	0,234	0,232	0,487	0,434	0,337	0,107
GCH	0,624	0,324	0,639	**0,640**	0,315	0,338	0,437	0,640	0,437	0,488	0,147
HARACOLOR	0,431	0,342	**0,475**	0,424	0,280	0,306	0,355	0,424	0,463	0,389	0,070
HARAFULL	0,520	0,346	0,402	**0,520**	0,290	0,304	0,295	0,520	0,426	0,402	0,099
HARALICK	0,364	0,285	0,334	0,458	0,289	0,283	0,277	0,457	**0,463**	0,357	0,082
JCD	**0,679**	0,303	0,432	0,434	0,345	0,361	0,415	0,434	0,434	0,426	0,106
LBP	0,257	0,185	0,381	**0,436**	0,162	0,173	0,171	0,436	0,434	0,293	0,126
LCH	**0,626**	0,342	0,437	0,433	0,511	0,371	0,624	0,433	0,437	0,468	0,101
MOMENTS	0,454	0,285	**0,471**	0,459	0,250	0,271	0,270	0,460	0,457	0,375	0,101
MPO	0,408	0,271	0,363	0,466	0,275	0,273	0,260	**0,468**	0,453	0,360	0,091
MPOC	0,393	0,294	**0,463**	0,420	0,257	0,276	0,354	0,421	0,448	0,369	0,077
PHOG	**0,478**	0,301	0,436	0,437	0,374	0,261	0,450	0,437	0,437	0,401	0,074
RCS	0,488	0,334	0,810	0,470	0,273	0,320	0,449	0,470	*0,811	0,492	0,196
TAMURA	**0,549**	0,259	0,435	0,436	0,284	0,271	0,539	0,436	0,435	0,405	0,110
Mean	0,504	0,312	0,487	0,466	0,301	0,301	0,393	0,466	0,481	-	

**Table 12:** Accuracy results obtained by each descriptor and clustering method from the dataset *D*_4_. The bold values correspond to the best clustering methods for each descriptor. The underlined values highlight the descriptor that reached the best performance for each clustering algorithm. The asterisk value represents the best combination (descriptor and clustering algorithm). It is also presented the mean accuracies considering all descriptors and clustering methods.

–	AGNES	CLARANS	CURE	DBSCAN	FCM	KMEANS	KMEDOIDS	OPTICS	ROCK	Mean Acc.	Std
ACC	0,549	0,391	**0,798**	0,575	0,371	0,374	0,587	0,576	0,784	0,556	0,161
BIC	**0,503**	0,366	0,478	0,429	0,328	0,370	0,418	0,429	0,435	0,417	0,055
CEDD	**0,740**	0,347	0,436	0,433	0,366	0,369	0,476	0,433	0,437	0,448	0,117
FCTH	0,682	0,402	**0,784**	0,431	0,370	0,470	0,483	0,431	0,433	0,498	0,139
GABOR	0,352	0,287	0,379	0,495	0,250	0,265	0,251	**0,503**	0,441	0,358	0,103
GCH	0,623	0,357	0,639	**0,640**	0,334	0,415	0,437	0,640	0,437	0,502	0,131
HARACOLOR	**0,504**	0,385	0,476	0,425	0,328	0,368	0,409	0,425	0,469	0,421	0,056
HARAFULL	**0,597**	0,368	0,532	0,487	0,327	0,349	0,320	0,486	0,426	0,432	0,099
HARALICK	0,465	0,356	0,381	0,454	0,309	0,326	0,319	0,454	**0,471**	0,393	0,068
JCD	**0,684**	0,524	0,431	0,434	0,350	0,402	0,415	0,434	0,434	0,456	0,097
LBP	0,407	0,229	0,380	**0,436**	0,185	0,201	0,194	0,436	0,434	0,322	0,116
LCH	**0,633**	0,393	0,437	0,433	0,521	0,390	0,624	0,433	0,437	0,478	0,093
MOMENTS	**0,487**	0,401	0,472	0,406	0,316	0,319	0,336	0,419	0,474	0,403	0,067
MPO	0,441	0,320	0,417	0,466	0,304	0,308	0,302	0,466	**0,482**	0,389	0,079
MPOC	0,396	0,261	0,424	0,423	0,299	0,322	0,315	0,423	**0,455**	0,369	0,070
PHOG	**0,528**	0,325	0,436	0,437	0,391	0,291	0,451	0,437	0,437	0,415	0,071
RCS	0,491	0,462	0,810	0,468	0,329	0,366	0,455	0,468	*0,811	0,518	0,174
TAMURA	**0,549**	0,395	0,436	0,436	0,314	0,316	0,539	0,436	0,436	0,428	0,082
Mean	0,535	0,365	0,508	0,461	0,333	0,346	0,407	0,463	0,485	-	

**Table 13:** Accuracy results obtained by each descriptor and clustering method from the dataset *D*_5_. The bold values correspond to the best clustering methods for each descriptor. The underlined values highlight the descriptor that reached the best performance for each clustering algorithm. The asterisk value represents the best combination (descriptor and clustering algorithm). It is also presented the mean accuracies considering all descriptors and clustering methods.

–	AGNES	CLARANS	CURE	DBSCAN	FCM	KMEANS	KMEDOIDS	OPTICS	ROCK	Mean Acc.	Std
ACC	0,446	0,294	0,463	0,685	0,297	0,319	0,448	**0,686**	0,462	0,455	0,148
BIC	0,448	0,314	0,829	0,829	0,308	0,331	0,444	0,829	**0,830**	0,574	0,248
CEDD	0,506	0,316	**0,831**	0,829	0,289	0,384	0,315	0,829	0,829	0,570	0,254
FCTH	0,557	0,355	0,468	0,827	0,444	0,448	0,318	0,828	**0,829**	0,564	0,209
GABOR	0,391	0,288	0,451	0,806	0,292	0,302	0,301	**0,809**	0,673	0,479	0,223
GCH	0,783	0,300	0,612	0,445	0,291	0,322	0,420	0,445	**0,830**	0,494	0,203
HARACOLOR	0,329	0,304	0,295	0,661	0,302	0,301	0,301	**0,673**	0,303	0,385	0,160
HARAFULL	0,605	0,425	0,480	0,804	0,387	0,429	0,359	0,814	**0,820**	0,569	0,195
HARALICK	0,485	0,328	0,442	0,800	0,314	0,310	0,310	**0,807**	0,636	0,492	0,207
JCD	0,505	0,433	0,829	**0,830**	0,296	0,416	0,356	0,830	0,829	0,592	0,233
LBP	0,687	0,331	0,749	0,826	0,308	0,347	0,308	**0,827**	0,824	0,579	0,246
LCH	0,654	0,310	0,830	0,829	0,351	0,358	0,459	0,829	*0,831	0,606	0,234
MOMENTS	0,351	0,308	0,451	0,730	0,305	0,306	0,306	**0,731**	0,507	0,444	0,178
MPO	0,474	0,341	0,478	0,792	0,303	0,304	0,305	0,805	**0,826**	0,514	0,230
MPOC	0,488	0,298	0,499	0,532	0,344	0,357	0,401	**0,535**	0,502	0,439	0,090
PHOG	0,739	0,353	0,830	0,825	0,349	0,355	0,420	0,826	*0,831	0,614	0,235
RCS	0,459	0,317	**0,478**	0,387	0,322	0,376	0,360	0,388	**0,478**	0,396	0,062
TAMURA	0,801	0,350	0,829	0,829	0,277	0,274	0,414	0,829	**0,830**	0,604	0,264
Mean	0,539	0,331	0,602	0,737	0,321	0,347	0,364	0,740	0,704	-	

The bold values highlight the clustering methods that obtained the best result for each descriptor. In addition, the underlined values emphasize the descriptor that reached the best performance for each clustering method. Finally, the asterisk value represents the best combination (descriptor and clustering method).

It is possible to observe that CURE and ROCK algorithms stand out for most datasets and descriptors, presenting the best results. The AGNES algorithm also reached good results. However, AGNES presented lower accuracy values compared to CURE and ROCK algorithms.

According to the obtained results, no descriptor can be considered the best for all clustering methods. However, in general, FCTH, GCH, and RCS descriptors presented the best accuracy values, especially when performed with AGNES, CURE, and ROCK clustering algorithms.

Since the internal and external samples are from the same cluster considering the *D*_5_ dataset, it is possible to observe a different behavior compared to other datasets. For instance, we notice that the OPTICS and ROCK algorithms present the highest accuracy for several descriptors. ROCK presents the best result, with an accuracy of 83.1%.

Since the internal and external samples are from the same cluster considering the *D*_5_ dataset, it is possible to observe a different behavior compared to other datasets. For instance, the OPTICS and ROCK algorithms present the highest accuracy for several descriptors. ROCK presents the best result, with an accuracy of 83.1%.

In addition to the accuracies, performance analyses were performed using other metrics (FOWLKES, DAVIES e CALINSKI). Tables 14–18 present the results obtained for each descriptor, considering the datasets *D*_1_ and *D*_2_ with CURE and the datasets *D*_3_, *D*_4_ and *D*_5_ with ROCK, since these were the best combinations (descriptor and clustering algorithm pair). In bold are highlighted the best results (descriptors) obtained according to each metric.

**Table 14:** Results obtained by each descriptor and clustering method CURE, considering each of the metrics (accuracy, FOWLKES, DAVIES and CALINSKI), from the dataset *D*_1_. Bold values highlight the best descriptors’ results according to each metric.

Descriptor	Accuracy	FOWLKES	DAVIES	CALINSKI
ACC	0,71	0,70	0,76	459,62
BIC	0,74	0,74	0,61	272,83
CEDD	0,74	0,71	0,67	266,75
FCTH	0,73	0,70	0,59	661,17
**GABOR**	0,47	0,47	**0,42**	2555,35
**GCH**	**0,77**	**0,78**	0,65	214,99
**HARACOLOR**	0,59	0,59	0,46	**12682,76**
HARAFULL	0,65	0,63	0,74	2668,87
HARALICK	0,53	0,55	0,43	3995,41
JCD	0,73	0,70	0,64	399,04
LBP	0,34	0,37	0,64	475,86
LCH	0,39	0,55	0,79	2,55
MOMENTS	0,72	0,73	0,65	2713,11
MPO	0,60	0,60	0,46	8487,48
MPOC	0,72	0,69	0,52	325,35
PHOG	0,40	0,55	0,71	5,98
RCS	0,72	0,73	0,59	2305,36
TAMURA	0,39	0,55	0,53	6,76

**Table 15:** Results obtained by each descriptor and clustering method CURE, considering each of the metrics (accuracy, FOWLKES, DAVIES and CALINSKI), from the dataset *D*_2_. Bold values highlight the best descriptors’ results according to each metric.

Descriptor	Accuracy	FOWLKES	DAVIES	CALINSKI
ACC	0,85	0,85	0,81	227,48
BIC	0,92	0,92	0,56	288,00
CEDD	0,92	0,92	0,93	132,14
**FCTH**	**0,93**	**0,92**	0,80	239,81
**GABOR**	0,70	0,74	**0,42**	2339,05
GCH	0,92	0,91	0,50	182,93
HARACOLOR	0,63	0,71	0,44	5414,58
HARAFULL	0,68	0,75	0,63	2047,40
HARALICK	0,63	0,64	0,44	2266,76
JCD	0,92	0,91	0,98	182,74
LBP	0,41	0,43	0,56	486,93
LCH	0,51	0,66	0,61	3,14
MOMENTS	0,68	0,75	0,56	2532,23
**MPO**	0,58	0,67	0,50	**5585,58**
MPOC	0,80	0,72	0,54	171,34
PHOG	0,51	0,65	0,78	5,27
RCS	0,92	0,92	0,63	868,74
TAMURA	0,51	0,66	0,58	4,79

**Table 16:** Results obtained by each descriptor and clustering method ROCK, considering each of the metrics (accuracy, FOWLKES, DAVIES and CALINSKI), from the dataset *D*_3_. Bold values highlight the best descriptors’ results according to each metric.

Descriptor	Accuracy	FOWLKES	DAVIES	CALINSKI
ACC	0,78	0,75	0,93	185,74
BIC	0,43	0,59	0,47	4,22
CEDD	0,44	0,59	1,05	1,17
FCTH	0,43	0,59	0,61	2,77
GABOR	0,43	0,42	0,39	2125,31
GCH	0,44	0,59	0,52	3,71
**HARACOLOR**	0,46	0,51	0,40	**8150,98**
HARAFULL	0,46	0,54	0,68	4103,04
HARALICK	0,43	0,58	0,26	19,61
JCD	0,43	0,59	0,67	2,43
LBP	0,43	0,59	0,76	22,62
LCH	0,44	0,59	1,05	0,93
MOMENTS	0,46	0,52	0,65	4752,47
MPO	0,45	0,49	0,41	7668,71
MPOC	0,45	0,47	0,57	1379,66
PHOG	0,44	0,59	0,45	4,65
**RCS**	**0,81**	**0,81**	0,45	1268,49
**TAMURA**	0,44	0,59	**0,37**	7,89

**Table 17:** Results obtained by each descriptor and clustering method ROCK, considering each of the metrics (accuracy, FOWLKES, DAVIES and CALINSKI), from the dataset *D*_4_. Bold values highlight the best descriptors’ results according to each metric.

Descriptor	Accuracy	FOWLKES	DAVIES	CALINSKI
ACC	0,78	0,75	1,23	238,77
BIC	0,44	0,59	0,47	4,34
CEDD	0,44	0,59	1,02	1,30
FCTH	0,43	0,59	0,61	2,76
GABOR	0,44	0,43	0,40	2624,93
GCH	0,44	0,59	0,51	3,93
HARACOLOR	0,47	0,56	0,37	5585,53
HARAFULL	0,47	0,55	0,65	4675,58
HARALICK	0,43	0,58	0,26	19,61
JCD	0,43	0,59	0,63	2,72
LBP	0,43	0,59	0,81	29,00
LCH	0,44	0,59	0,85	1,44
MOMENTS	0,47	0,56	0,60	4486,74
**MPO**	0,48	0,53	0,38	**6614,24**
MPOC	0,46	0,48	0,58	1607,25
PHOG	0,44	0,59	0,44	4,85
**RCS**	**0,81**	**0,81**	0,46	1629,90
**TAMURA**	0,44	0,59	**0,35**	8,64

**Table 18:** Results obtained by each descriptor and clustering method ROCK, considering each of the metrics (accuracy, FOWLKES, DAVIES and CALINSKI), from the dataset *D*_5_. Bold values highlight the best descriptors’ results according to each metric.

Descriptor	Accuracy	FOWLKES	DAVIES	CALINSKI
ACC	0,46	0,59	0,93	556,89
BIC	0,83	0,84	0,45	4,78
CEDD	0,83	0,83	0,74	1,76
FCTH	0,83	0,83	0,64	2,78
GABOR	0,67	0,68	0,34	1251,93
GCH	0,83	0,84	0,46	5,01
HARACOLOR	0,64	0,65	0,34	1107,21
**HARAFULL**	0,30	0,42	0,47	**7080,66**
HARALICK	0,82	0,82	0,26	19,61
JCD	0,83	0,83	0,55	3,19
LBP	0,82	0,83	0,37	67,47
**LCH**	**0,83**	**0,84**	0,94	1,18
MOMENTS	0,51	0,54	0,54	2663,56
MPO	0,83	0,83	0,41	15,28
MPOC	0,50	0,59	0,48	1332,25
PHOG	0,83	0,84	0,42	5,23
RCS	0,48	0,59	0,56	3197,07
**TAMURA**	0,83	0,84	**0,29**	12,21

It is possible to observe that the FOWLKES metric presents similar results to the accuracy. When DAVIES and CALINSKI metrics reach their best results (bold values), in most cases, accuracy values are inferior to the best accuracy obtained for the clustering. This may indicate that metrics based on cohesion and clustering separation, in this context, should not be used to analyze correctability.

**Fig 3:**
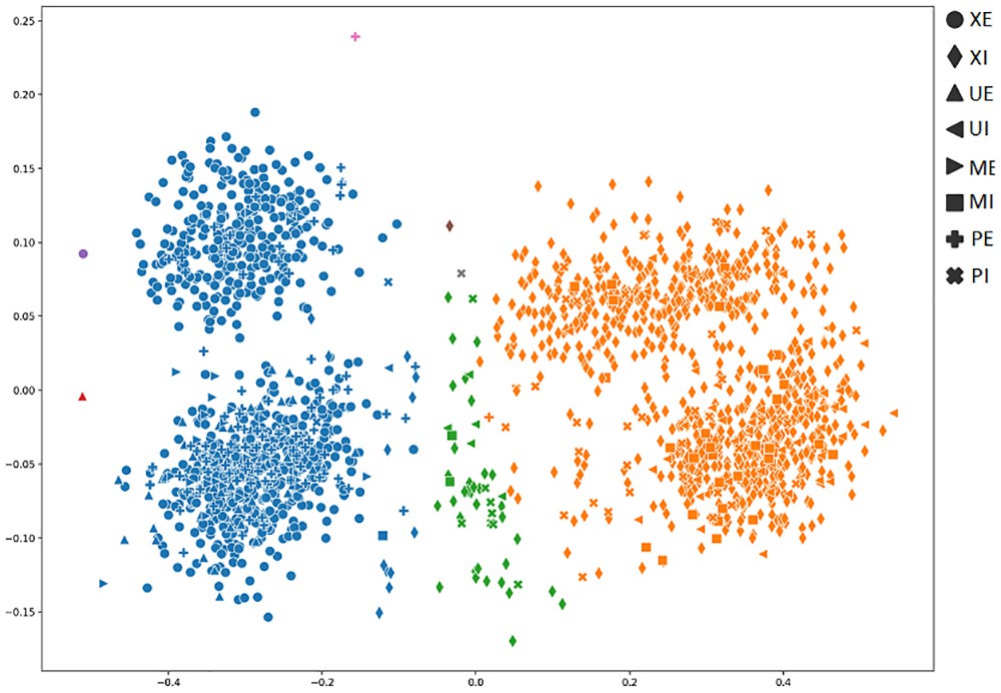
Clusterings’ visualization obtained by PCA from the *D*_4_ dataset, considering the best pair RCS_ROCK (descriptor_clustering algorithm), and according to the accuracy metric.

**Fig 4:**
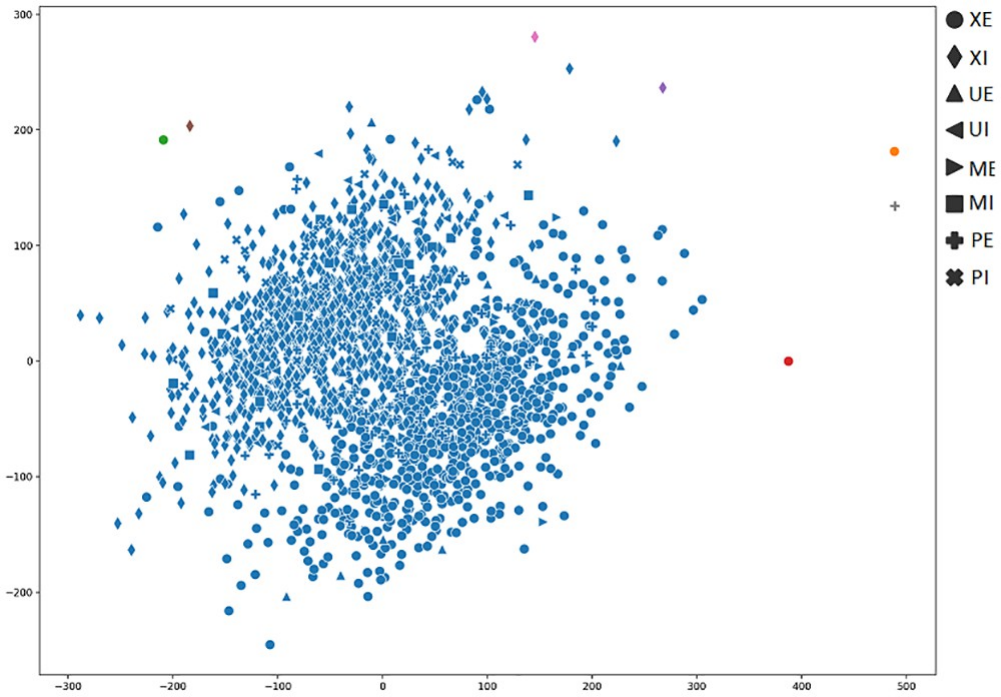
Clusterings’ visualization obtained by PCA from the *D*_4_ dataset, considering the best pair TAMURA_ROCK (descriptor_clustering algorithm), and according to the DAVIES metric.

**Fig 5:**
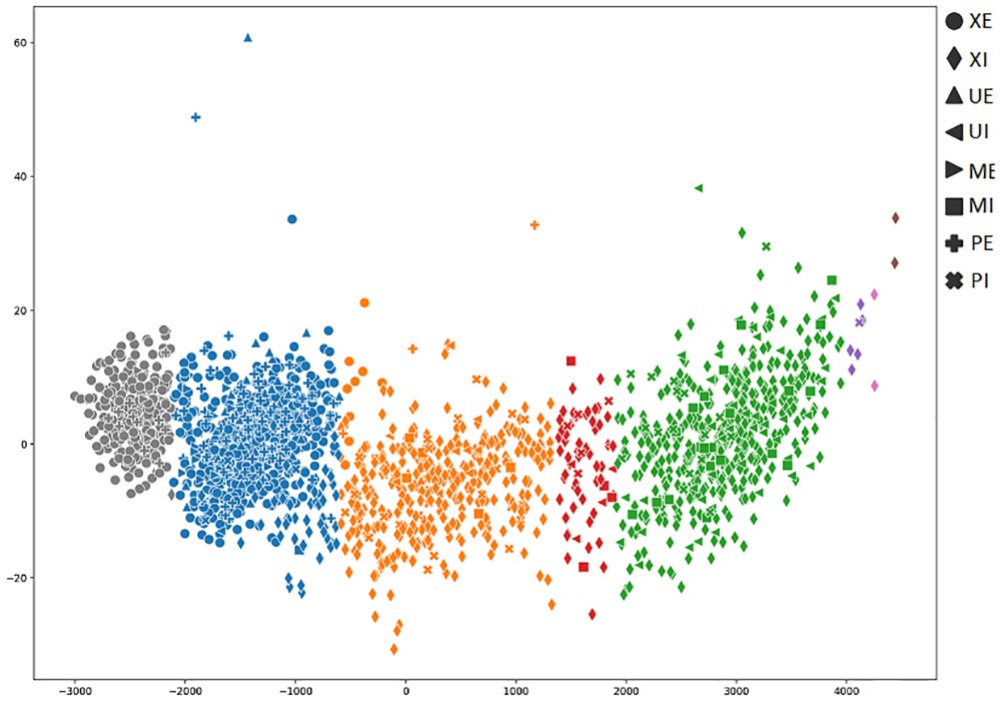
Clusterings’ visualization obtained by PCA from the *D*_4_ dataset, considering the best pair MPO_ROCK (descriptor_clustering algorithm), and according to the CALINSKI metric.

**Fig 6:**
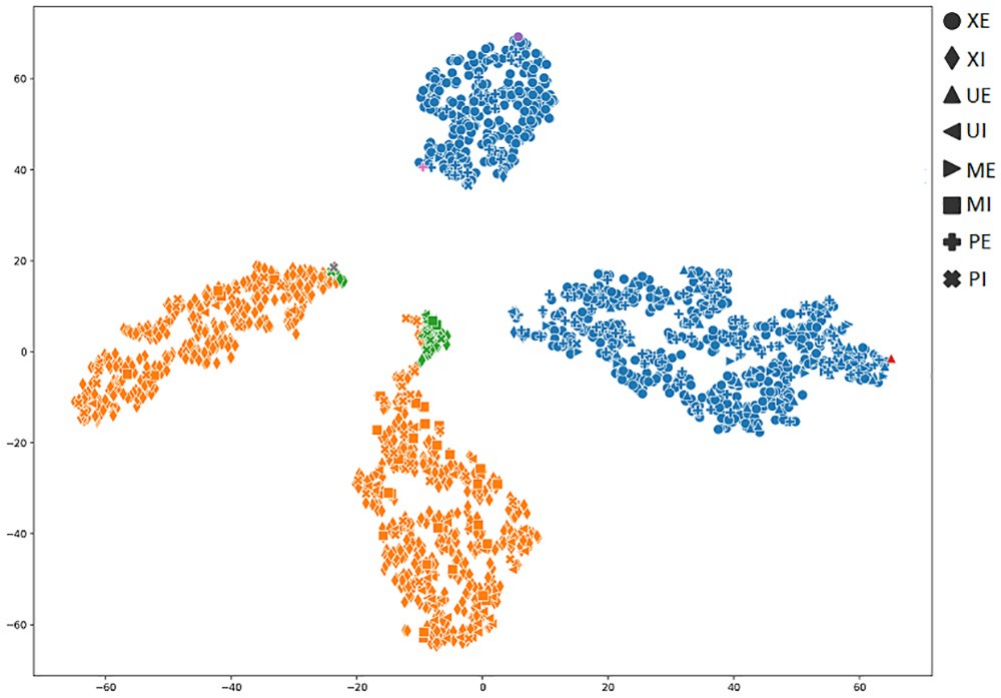
Clusterings’ visualization obtained by t-SNE from the *D*_4_ dataset, considering the best pair RCS_ROCK (descriptor_clustering algorithm), and according to the accuracy metric.

**Fig 7:**
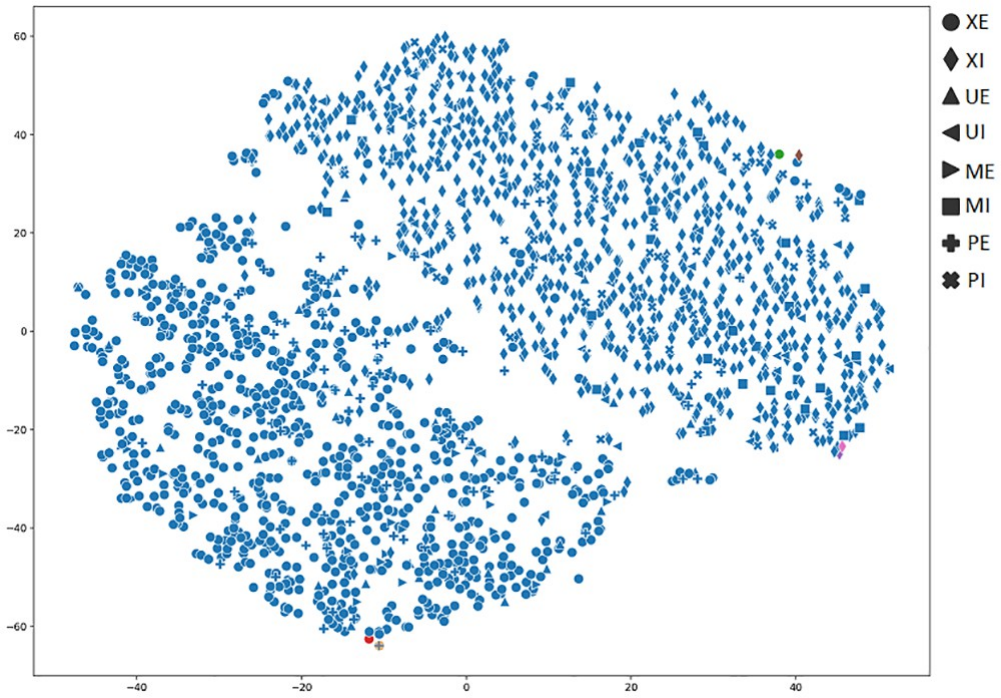
Clusterings’ visualization obtained by t-SNE from the *D*_4_ dataset, considering the best pair TAMURA_ROCK (descriptor_clustering algorithm), and according to the DAVIES metric.

**Fig 8:**
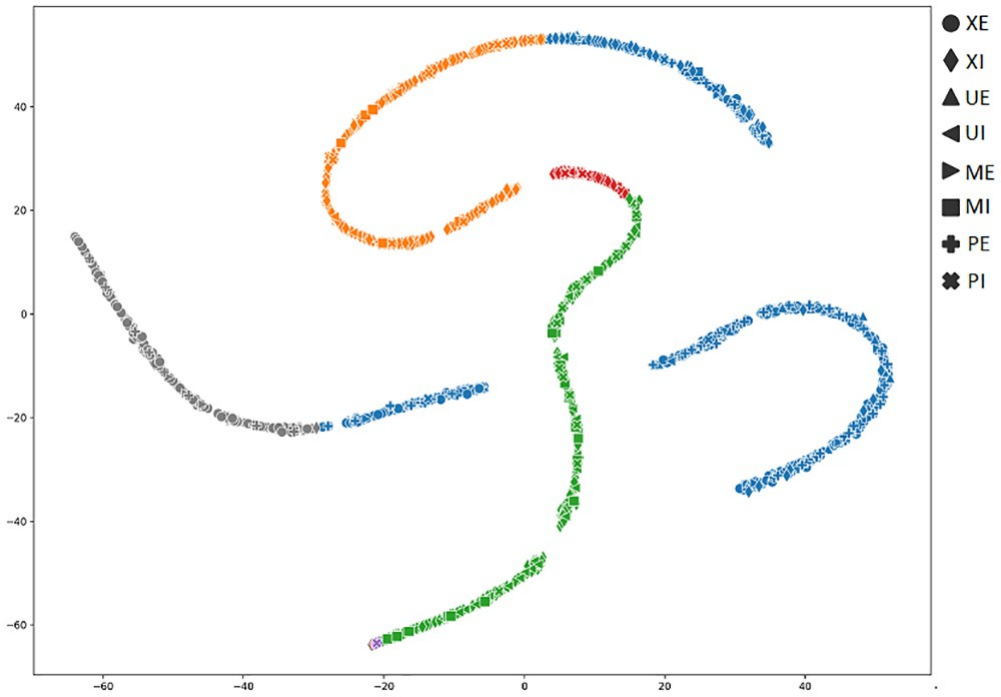
Clusterings’ visualization obtained by t-SNE from the *D*_4_ dataset, considering the best pair MPO_ROCK (descriptor_clustering algorithm), and according to the CALINSKI metric.

In order to achieve a better understanding of the results obtained by the several metrics, it is vital to observe the graphical clustering representation. To do so, PCA and t-SNE dimensionality reduction techniques were considered (Figs 3–8), respectively. Figs 3–8 present the highlighted clustering results from Table 17 (i.e., best combinations—descriptor and clustering algorithm—according to each metric). The colors represent an obtained clustering, while symbols represent the true classes of the samples.

As presented in Table 4, the majority (83%) of the dataset’s samples are from the perfect, external and internal classes. It is possible to observe the clustering of these classes of samples, represented by circle and diamond symbols (Figs 3–8)). Both PCA and t-SNE techniques could not correctly cluster other damage classes. In the clustering presented in Fig 3, even with an 81% of accuracy, it is possible to note that the majority of moisture, bugs, and mechanical damage classes were considered perfect, that is, almost all of the successes obtained refer to perfect classes.

When analyzing Fig 4, it is possible to conclude that the highlighted results for DAVIES metric (e.g., 0.35 value to the TAMURA_ROCK pair in Table 17), do not indicate a good clustering, since a cluster with almost all samples was generated. In this case, the accuracy obtained from the clustering was 44%. Therefore, DAVIES is not a good metric for this context.

We noticed this same behavior in Fig 5, where we can see that the results obtained from the CALINSKI metric (e.g., 6614.24 for the MPO_ROCK pair in Table 17), do not show a good clustering formation. The majority of samples from both perfect classes (i.e., XE and XI, represented by circle and diamond, respectively) that comprise almost 83% of the samples, were divided into five clusters, impacting the accuracy (48%). Therefore, CALINSKI is not a good metric for this context either.

## 5 Conclusion

In this paper, we performed an extensive experimental evaluation, considering different unsupervised learning techniques applied to soybean seed image datasets from the tetrazolium test. To do so, we used 5 image datasets considering different scenarios, damages, and/or their respective severity levels. To describe these images, we consider 18 different image descriptors. Moreover, we evaluated 9 clustering algorithms from different paradigms (e.g., partitional, hierarchical, and density-based) under 4 metrics (accuracy, FOWLKES, DAVIES, and CALINSKI). We considered 2 dimensionality reduction techniques (PCA and t-SNE) to validate the analyses to visualize the clusters’ distributions.

Analyzing the obtained results, we observed similar behavior with different datasets, in which the best accuracies were achieved according to the number of perfect samples (without damage) in the dataset. The samples’ unbalancing in each class, including the excess of perfect samples, disturbs the clustering algorithm’s performance, especially in classes with few samples.

We generally reached the best accuracy results by each descriptor with AGNES, CURE, and ROCK clustering algorithms. FCTH, GCH, and RCS presented the highest accuracy values among all descriptors evaluated, using the combination FCTH_CURE, GCH_CURE, and RCS_ROCK.

Regarding the generated visualizations, it was possible to observe that the FOWLKES metric presented results similar to the accuracy; this suggests that the metric can be used as a substitute for accuracy in clustering analysis.
